# Hepatitis B vaccine knowledge, uptake and associated factors among healthcare workers in public secondary facilities in Delta Central District, Nigeria

**DOI:** 10.4314/gmj.v59i3.6

**Published:** 2025-09

**Authors:** Okeroghene K Sajini, Maureen I Ntaji, Nyemike S Awunor, Mamodesan T Okumagba, Oghenetejiri P Sajini

**Affiliations:** 1 Community Medicine Department, Delta State University, Abraka, Nigeria; 2 Radiology Department, Federal Medical Centre, Asaba, Nigeria

**Keywords:** Hepatitis B, healthcare workers, health facilities, vaccination

## Abstract

**Objectives:**

The study determined the knowledge of Hepatitis B vaccine, vaccination status and associated factors among healthcare workers in public secondary health facilities in the Delta Central Senatorial District, Nigeria.

**Design:**

A cross-sectional study.

**Settings:**

Healthcare workers employed in public secondary health facilities in the Delta Central District.

**Participants:**

Four hundred and sixteen participants.

**Main outcome measures:**

Knowledge of the Hepatitis B vaccine, vaccination status, and predictors of vaccine uptake.

**Results:**

Majority of the participants (78.0%) had good knowledge about the Hepatitis B vaccine. One hundred and seventeen participants (42.6%) were fully vaccinated. Age, sex, marital status, education level, cadre, and knowledge of HBV were significantly associated with vaccination status (p < 0.05). Factors associated with low uptake included being female [adjusted odds ratio (AOR) 1.884; 95% CI 1.128-3.147], being a nurse (AOR 0.228; 95% CI: 0.071-0.737) and poor knowledge (AOR: 0.404; 95% CI: 0.184-0.889).

**Conclusion:**

This study showed a high level of knowledge about Hepatitis B vaccine, and a suboptimal vaccination rate among healthcare workers.

**Funding:**

No funding was acquired for the present research.

## Introduction

The Hepatitis B virus (HBV) causes Hepatitis B infection, a vaccine-preventable and potentially fatal liver disease. It is a significant global health issue that can result in persistent infection and greatly increase the risk of cirrhosis and liver cancer-related mortality. 1

Nigeria has a high prevalence of HBV, with medical professionals at higher risk of contracting the virus. 2 Healthcare workers in Nigeria, similar to those in other countries, are more susceptible to HBV infection due to occupational exposure to bodily fluids like blood. 3 The World Health Organisation reports that 90% of occupationally acquired Hepatitis B infections occur in low-income nations, primarily in Sub-Saharan Africa, with approximately two million healthcare workers at risk annually.^1^ Compared to the general public, health professionals are four times more likely to contract HBV.^4^ An estimated 20 million Nigerians have Hepatitis B or C, but over 80% of those affected remain unaware of their condition.^5^

There is evidence of vaccine hesitancy in Africa. A recent survey demonstrated a global decline in trust in vaccines between 2015 and 2019, including in Nigeria.^6^ Before the COVID-19 pandemic and vaccine release, vaccination receptivity was higher. Misconceptions regarding the origins, efficacy, and potential adverse effects of the COVID-19 vaccine have contributed to hesitancy. 7,8,9 In an economic context, Sim et al.^10^ estimate significant net benefits of immunisation against various pathogens for low and middle-income countries. Using the cost-of-illness and value-of-a-statistical-life methods, the net benefits of immunisation against 10 pathogens for 94 low and middle-income countries between 2011 and 2030 are estimated at $1,445.3 billion and $3,371.5 billion, respectively.

However, despite the proven benefits, vaccine reluctance remains a top global health threat, highlighting the need for a more comprehensive understanding of the socio-cultural and behavioural factors that affect immunisation programmes.^1^,^4^

This study aimed to determine the knowledge of the Hepatitis B vaccine, Hepatitis B vaccine status, and associated factors among healthcare workers in public secondary health facilities in the Delta Central Senatorial District, Nigeria.

## Methods

### Study area

This research was conducted in the Delta Central Senatorial District, Delta State. There are eight Local Government Areas in the Delta Central Senatorial District. They are: Ethiope East, Ethiope West, Sapele, Okpe, Ughelli North, Ughelli South, Udu and Uvwie. In the Delta Central Senatorial District, Delta State, Nigeria, there are nineteen public secondary health facilities.

### Study design

The study was a cross-sectional design conducted to assess the knowledge of Hepatitis B vaccine, vaccination status and associated factors among healthcare workers (HCWs) in public secondary health facilities within Delta Central Senatorial District, Delta State, Nigeria.

### Study population

The study population was healthcare workers (HCWs), male and female, from selected public secondary health facilities in Delta Central District, Delta State, Nigeria. The category of HCWs recruited included doctors, nurses, laboratory scientists/technicians, pharmacists/pharmacy technicians, optometrists, radiographers, and other categories of HCWs such as health assistants, cleaners and mortuary attendants.

### Sample size estimation

The minimum sample size of 383 was estimated using the Cochrane formula n = Z2p(1-p)/d2 with a prevalence of 48.5% from a previous study among HCWs in Lagos, Nigeria.^11^ The confidence interval was 95% and the margin of error was set at 5%. An attrition rate of 10% was envisaged. The estimated sample size was 422.

### Sampling technique

A multi-stage probability sampling was used to select study participants. First, simple random sampling was used to randomly select five from eight Local Government Areas in the Delta Central Senatorial District by balloting. The next step involved selecting one public secondary health facility from each of the five Local Government Areas in the previous step through a simple ballot. Finally, data were collected from the HCWs in each selected health facility using purposive sampling, ensuring representation across various healthcare professions.

### Data collection

#### Questionnaire

Data were collected from consenting healthcare workers (HCWs) using a self-administered questionnaire. The questionnaire was pre-tested in a health facility in the Delta North Senatorial District, Delta State, Nigeria. The questionnaire had three sections. Section A contained socio-demographic characteristics of HCWs. Section B assessed knowledge of HCWs regarding the Hepatitis B vaccine. Section C focused on vaccination status.

### Ethical consideration

Ethical approval was obtained from Delta State University Teaching Hospital Health Research Ethics Committee with approval number HREC/PAN/2023/082/0606. Informed consent was obtained from all study participants.

### Data analysis

The Statistical Package for Social Sciences (SPSS, v22) was used to analyse the data obtained from the study. Simple descriptive and inferential statistics were conducted. Participants' knowledge of Hepatitis B and vaccination was assessed through questions about transmission, general knowledge of the vaccine, and side effects and contraindications. Each question had a correct answer and multiple wrong answers. Each correct answer was scored as one, while each wrong choice was scored as zero. The overall score was converted to a percentage. Those who scored below 50% were considered to have poor knowledge, while those who scored 50% and above were considered to have good knowledge.

Participants were categorised into two groups based on their vaccination status. Those who had received the required three doses were considered to have good uptake (optimal), while those who received fewer than three doses were considered to have poor uptake (suboptimal). Tests of significance were performed using a 95% confidence interval, with a significance level set at P < 0.05. Logistic regression was used to determine socio-demographic variables and other variables that were independently associated with HBV vaccine knowledge and HBV vaccine status.

## Results

### General characteristics

Only 416 questionnaires were completed and returned. The socio-demographic characteristics of the participants are presented in Table 1. The study involving 416 secondary public healthcare personnel revealed a diverse demographic profile. The largest proportion of participants were 30-39 years old, female, married, and highly educated, with 78.1% holding tertiary degrees.

**Table 1:** Socio-demographic Characteristics of the Study Participants (n = 416)

		Frequency, n (%)
**Age**	20-29	105 (25.2)
	30-39	170 (40.9)
	40-49	89 (21.4)
	50 and above	52 (12.5)
	Mean (SD)	37 (±14)

**Gender**	Male	141 (33.9)
	Female	275 (66.1)


**Marital status**	Single	162 (38.9)
	Married	219 (52.6)
	Divorced	21 (5.1)
	Widowed	14 (3.4)

**Ethnic group**	Urhobo	215 (51.7)
	Itsekiri	45 (10.8)
	Ijaw	46 (11.0)
	Isoko	54 (13.0)
	*Others	56 (13.5)

**Level of education**	Primary	10 (2.4)
	Secondary	66 (15.9)
	Tertiary	324 (77.9)
	None	16 (3.8)

**Cadre**	Medical doctor	58 (13.9)
	Nurse	108 (26.0)
	Laboratory Scientist/Technician	44 (10.6)
	Pharmacist/Pharmacy Technician	48 (11.5)
	Physiotherapist	17 (4.1)
	Radiographer	13 (3.1)
	Health Assistant	82 (19.7)
	Cleaner	26 (6.3)
	**Others	20 (4.8)

* Others: Ukwuani, Igbo and Yoruba

** Others: Optometrists, Dentists, Mortuary attendants etc.

The survey represented various ethnic groups and healthcare professionals, including doctors, nurses, and laboratory technicians.

### Knowledge of Hepatitis B vaccine among healthcare workers

The respondents showed good overall knowledge, 326 (78.0%), with 351 (84.4%) understanding the transmission, and 354 (85.1%) admitting the vaccine's recommendation for healthcare workers. However, only 79 (19.0%) correctly identified the population at risk. A significant proportion of respondents believed that the HBV vaccine provides lifelong immunity 308, 73.1%) and prevents liver cancer 280, 67.3%). Knowledge of the side effects 243 (58.4%) and precautions/contraindications 115 (27.6%) of the HBV vaccine was relatively low.

### Hepatitis B Vaccination Status among Participants

**Figure 1:**
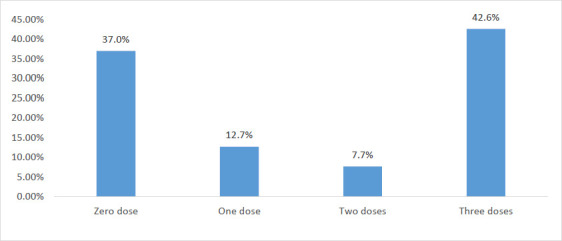
Number of HBV vaccine doses received by healthcare workers who were vaccinated (n = 416)

The majority of participants, 262 (63.0%), had received at least one dose of the Hepatitis B vaccine, with 177 (42.6%) receiving the required three doses. However, 154 (37.0%) had not received any dose, as shown in Figure 1.

### Association between the uptake of HBV vaccine, socio-demographic factors and level of knowledge

As shown in Table 3, a significant association was found between age group (p = 0.035), sex (p = 0.012), marital status (p = 0.014), level of education (p < 0.001), cadre (p < 0.001), knowledge of Hepatitis B vaccine (p < 0.001) and HBV vaccination status.

**Table 3:** Relationship between the level of uptake of HBV, socio-demographic parameters and level of knowledge of the study participants

		Optimal Uptake	Suboptimal Uptake	χ^2^	p-value
		n (%)	n (%)		
**Knowledge Level**	**Good**	**167 (94.4)**	**159 (66.5)**	**81.533**	**<0.001**
	**Poor**	**10 (5.6)**	**80 (33.5)**		
**Age (years)**	**20 – 29**	**34 (19.2)**	**71 (29.7)**	**8.617**	**0.035**
	**30 – 39**	**79 (44.6)**	**91 (38.1)**		
	**40 – 49**	**45 (25.4)**	**44 (18.4)**		
	**≥ 50**	**19 (10.7)**	**33 (13.8)**		
**Sex**	**Male**	**72 (40.7)**	**69 (28.9)**	**6.328**	**0.012**
	**Female**	**105 (59.3)**	**170 (71.1)**		
**Marital Status**	**Single**	**57 (32.4)**	**105 (43.9)**	**10.635**	**0.014**
	**Married**	**103 (58.5)**	**116 (48.5)**		
	**Divorced**	**13 (7.4)**	**8 (3.3)**		
	**Widowed**	**3 (1.7)**	**10 (4.2)**		
**Level of Education**	**None**	**1 (0.6)**	**14 (5.9)**	**26.214**	**<0.001**
	**Primary**	**2 (1.1)**	**8 (3.4)**		
	**Secondary**	**15 (8.5)**	**51 (21.4)**		
	**Tertiary**	**159 (89.8)**	**165 (69.3)**		
**Cadre**	**Doctor**	**41 (23.2)**	**17 (7.1)**	**46.082**	**<0.001**
	**Nurse**	**54 (30.5)**	**54 (22.6)**		
	**Lab. Scientist**	**21 (11.9)**	**23 (9.6)**		
	**Pharmacist**	**21 (11.9)**	**27 (11.3)**		
	**Physiotherapist**	**7 (4.0)**	**10 (4.2)**		
	**Radiographer**	**3 (1.7)**	**10 (4.2)**		
	**Health Assistant**	**24 (13.6)**	**58 (24.3)**		
	**Support Staff**	**6 (3.4)**	**40 (16.7)**		

### Socio-demographic factors as possible predictors of the level of knowledge among respondents

Table 4 shows the associated factors. Although not statistically significant, the level of uptake increases with age, with those ≥ 50 years about two times more likely (AOR: 1.845; 95% CI: 0.719 - 4.735) to take the vaccine compared to the referenced age group (20-29 years). Also, females were significantly more likely to take up the vaccines by about two times (AOR: 1.884; 95% CI: 1.128 – 3.147) compared to males. Regarding the marital statuses, with reference to singles, those who were married were most likely to take up the vaccines (AOR: 2.908; 95% CI: 0.967 – 8.748) while the widows were twice less likely to take up the vaccines (AOR: 0.533; 95% CI: 0.114 – 2.490).

**Table 4:** Socio-demographic factors as possible predictors of the level of knowledge among respondents

		B	S.E.	Sig.	Exp(B)	95% C.I. for EXP(B)	

						Lower	Upper
**Age**	**20 – 29**			0.471	1.000		
	**30 – 39**	-0.046	0.322	0.885	0.955	0.508	1.793
	**40 – 49**	0.160	0.405	0.693	1.173	0.531	2.594
	**≥ 50**	0.613	0.481	0.203	1.845	0.719	4.735
**Gender**	**Male**				1.000		
	**Female**	0.634	0.262	0.015	1.884	1.128	3.147
**Marital Status**	**Single**			0.006	1.000		
	**Married**	1.067	0.562	0.057	2.908	0.967	8.748
	**Divorced**	0.222	0.558	0.691	1.249	0.418	3.728
	**Widowed**	-0.630	0.787	0.423	0.533	0.114	2.490
**Level of Education**	**None**			0.050	1.000		
	**Primary**	1.779	0.732	0.015	5.922	1.412	24.845
	**Secondary**	1.265	0.704	0.072	3.542	0.892	14.073
	**Tertiary**	2.712	1.276	0.034	15.061	1.236	183.600
**Cadre of health workers**	**Doctor**			0.128	1.000		
	**Nurse**	-1.478	0.598	0.013	0.228	0.071	0.737
	**Lab. Scientist**	-0.946	0.544	0.082	0.388	0.134	1.128
	**Pharmacist**	-0.352	0.599	0.557	0.704	0.218	2.274
	**Physiotherapist**	-0.569	0.596	0.34	0.566	0.176	1.821
	**Radiographer**	-0.948	0.734	0.196	0.387	0.092	1.633
	**Health Assistant**	-0.152	0.884	0.864	0.859	0.152	4.858
	**Support Staff**	-0.405	0.538	0.452	0.667	0.232	1.916
**Level of knowledge**	**Good**			0.000	1.000		
	**Poor**	-0.905	0.402	0.024	0.404	0.184	0.889

Increasing level of education also increased the likelihood of taking up the vaccines; those with primary education were five times more likely (AOR: 5.922; 95% CI: 1.412 – 24.845), those with secondary education were three times more likely (AOR: 3.542; 95% CI: 0.892 – 14.073) and those with tertiary education being fifteen times more likely (AOR: 15.061; 95% CI: 1.236 – 183.600).

Regarding the cadre of the healthcare workers, while doctors were the most likely to receive the vaccine, the health assistants were next, while nurses were the least likely to take up the vaccine (AOR: Nurse 0.228; 95% CI: 0.071 – 0.737). Finally, those with poor knowledge were about six times less likely (AOR: 0.404; 95% CI: 0.184 – 0.889) to take up the vaccine.

## Discussion

### General characteristics

Of the total respondents, 66.1% were female. This suggests that a significant majority of the respondents were women. A possible reason for this gender distribution is that certain professions, such as nursing and health assistants, are often more commonly associated with women than men. This could have influenced the higher representation of females in the survey. The majority of respondents were in the nursing and health assistant cadre. The largest proportion (78.1%) of the respondents had a tertiary education, indicating a relatively high level of education among the surveyed individuals.

Three point eight per cent (3.8%) of the respondents had no formal education, which is a small minority. This implies that the majority of the respondents were well-educated. The high level of education among respondents is seen as an advantage because it likely enabled them to understand the survey questions easily. Respondents with higher education levels may have better comprehension and the ability to provide more detailed and accurate responses.

### Knowledge about Hepatitis B vaccine

This study demonstrates a good level of knowledge about the Hepatitis B vaccine among HCWs.

At a 78.0% knowledge level, this result is comparable to those obtained in some studies conducted in Nigeria. Oni et al 12 found that 89.2% of respondents had good knowledge about the Hepatitis B vaccine.

This level of knowledge may have been influenced by increasing activities of non-governmental organisations (NGOs) in creating awareness about Hepatitis B infection. However, significant knowledge gaps still exist, particularly regarding risk groups and vaccine efficacy. In comparison, the proportion of knowledge in this study is higher than what was obtained by Bedaso et al.^4^ in Ethiopia, who reported that only 49.8% of respondents had good knowledge about the Hepatitis B vaccine. These differences highlight the role of institutional policies in promoting vaccine literacy among HCWs.

### Vaccination status

This study revealed a notable disparity in Hepatitis B vaccination rates among respondents, with 63% having received at least one dose of the vaccine. However, a more alarming trend emerged, as only 42.6% of those who initiated vaccination completed the full dose. This indicates a suboptimal vaccination coverage, highlighting the need for improved vaccine administration and follow-through.

Comparatively, our findings align with existing literature, which also indicates inadequate Hepatitis B vaccination coverage among healthcare workers. For instance, Kooffreh-Ada et al. 13 reported a slightly lower vaccination rate, with 43.2% of participants completing the three-dose regimen, surpassing the completion rate observed in this study. Conversely, Oni et al. 12 found a marginally lower initiation rate, with 41.5% of respondents receiving at least one dose.

A more striking contrast emerges when considering the findings of Dayyab et al. 14, which revealed a significantly lower vaccination coverage, with only 18.1% of participants completing the full three-dose regimen. This disparity underscores the variability in vaccination rates across different populations and settings.

The factors associated with suboptimal uptake, such as being female, having lower education levels, or belonging to specific cadres like nursing, suggest that socio-demographic and occupational factors play a significant role in vaccination behaviour. For instance, the finding that nurses are the least likely to be vaccinated, despite their high exposure to occupational risks, warrants further investigation, particularly regarding workload constraints, institutional vaccination policies, or misconceptions about the safety of the vaccine.

Additionally, female HCWs were nearly twice as likely as males to be vaccinated, which may reflect gender differences in health-seeking behaviour. This finding is consistent with global trends showing that women are generally more proactive about preventive healthcare.

Addressing the barriers to Hepatitis B vaccination uptake requires targeted workplace vaccination programs, enhanced educational campaigns, policy interventions to improve vaccine accessibility, and efforts to build trust in vaccines. By strengthening the Hepatitis B vaccination rate among healthcare workers, we can reduce the spread of Hepatitis B in healthcare settings and protect both healthcare workers and the communities they serve.

A limitation of this study was the reliance on self-reported vaccination status, which introduces the possibility of reporting bias, as respondents may wrongly report their vaccination history. Additionally, the study did not verify vaccination claims through medical records, which could lead to inaccuracies in the data. Addressing this limitation in future research through verified medical documentation would enhance the reliability of findings.

## Conclusion

This study highlights the need for sustained efforts to improve Hepatitis B vaccination coverage among healthcare workers in Nigeria. While knowledge about the vaccine is high, vaccination rates remain suboptimal, particularly in terms of completing the full vaccination schedule, and with significant disparities based on education, profession, and gender. Further studies should concentrate on comparative studies to evaluate vaccination practices in other healthcare settings, and longitudinal studies to evaluate the long-term efficacy of vaccination programmes to healthcare workers.

## Figures and Tables

**Table 2:** Healthcare workers' knowledge of Hepatitis B virus vaccine (*n*=416)

Variables	Response	Frequency, n (%)
Hepatitis B transmission	Correct	351 (84.4)
	Incorrect	65 (15.6)
Infected without showing symptoms	Correct	276 (66.3)
	Incorrect	140 (33.7)
Population at higher risk of HBV infection	Correct	79 (19.0)
	Incorrect	337 (81.0)
HBV vaccine recommended for HCWs	Correct	354 (85.1)
	Incorrect	62 (14.9)
Required dose of HBV vaccine	Correct	230 (55.3)
	Incorrect	186 (44.7)
HBV vaccine provides lifelong immunity	Correct	308 (74.0)
	Incorrect	108 (26.0)
Repeat HBV vaccination	Correct	257 (61.8)
	Incorrect	159 (38.2)
HBV vaccine prevents liver cancer	Correct	280 (67.3)
	Incorrect	136 (32.7)
HBV vaccine has side effect	Correct	243 (58.4)
	Incorrect	173 (41.6)
HBV vaccine precaution/contraindication	Correct	115 (27.6)
	Incorrect	301 (72.4)
